# Translating evidence into practice in primary care management of adolescents and women with polycystic ovary syndrome: a mixed-methods study

**DOI:** 10.1093/fampra/cmae007

**Published:** 2024-03-04

**Authors:** Stephanie Cowan, Lisa Moran, Rhonda Garad, Elizabeth Sturgiss, Siew Lim, Carolyn Ee

**Affiliations:** Monash Centre for Health Research and Implementation, Monash University, Melbourne, Australia; Monash Centre for Health Research and Implementation, Monash University, Melbourne, Australia; Monash Centre for Health Research and Implementation, Monash University, Melbourne, Australia; School of Primary and Allied Health Care, Monash University, Melbourne, Australia; Monash Centre for Health Research and Implementation, Monash University, Melbourne, Australia; Health Systems and Equity, Eastern Health Clinical School, Monash University, Melbourne, Australia; NICM Health Research Institute, Western Sydney University, Sydney, Australia

**Keywords:** behaviour change, general practice, guideline, polycystic ovary syndrome, research translation

## Abstract

**Background:**

The international guideline on polycystic ovary syndrome (PCOS) provides evidence-based recommendations on the management of PCOS. Guideline implementation tools (GItools) were developed for general practitioner (GP) use to aid rapid translation of guidelines into practice. This mixed-methods study aimed to evaluate barriers and enablers of the uptake of PCOS GItools in general practice.

**Design and setting:**

A cross-sectional survey was distributed through professional networks and social media to GPs and GPs in training in Australia. Survey respondents were invited to contribute to semi-structured interviews. Interviews were audio-recorded and transcribed verbatim. Qualitative data were thematically analysed and mapped deductively to the Theoretical Domains Framework and Capability, Opportunity, Motivation and Behaviour model.

**Results:**

The study engaged 146 GPs through surveys, supplemented by interviews with 14 participants. A key enabler to capability was reflective practice. Barriers relating to opportunity included limited awareness and difficulty locating and using GItools due to length and lack of integration into practice software, while enablers included ensuring recommendations were relevant to GP scope of practice. Enablers relevant to motivation included co-use with patients, and evidence of improved outcomes with the use of GItools.

**Discussion:**

This study highlights inherent barriers within the Australian healthcare system that hinder GPs from integrating evidence for PCOS. Findings will underpin behaviour change interventions to assist GPs in effectively utilising guidelines in clinical practice, therefore minimising variations in care. While our findings will have a direct influence on guideline translation initiatives, changes at organisational and policy levels are also needed to address identified barriers.

**Key messages**

- Environmental restructuring is key to guideline implementation by GPs.
- This includes awareness raising and integration into practice software.
- Reflective practice may also aid guideline implementation.

## Introduction

Polycystic ovary syndrome (PCOS) is a common endocrine condition affecting 8-13% of reproductive-aged women.^1^ General practitioners (GPs) provide comprehensive, holistic, and continuous care for all people and are well-placed to provide care to women with PCOS. GPs play a vital role in timely and accurate diagnosis, provision of information and education, lifestyle and weight management, and management of key features of PCOS, such as hyperandrogenism, emotional wellbeing, infertility, and cardiometabolic risk. As providers of first-contact care and coordinators of care within a multidisciplinary team, GPs are essential in the management of PCOS.^2^

The 2018 international guideline on PCOS provided evidence-based recommendations on diagnosis and management of PCOS and aimed to minimise variations in care.^1^ However, clinical practice guidelines remain underused,^3^ and time to implementation averages 15 years.^4^ Guideline implementation tools (GItools) are resources, strategies or materials (e.g. algorithms, concise summaries) designed to facilitate implementation of guideline recommendations. GItools aim to bridge the gap between recommendations and application in real-world settings by overcoming barriers to use and enhancing healthcare professionals’ ability to implement recommendations^,^.^5^ There is evidence that guidelines with accompanying GItools are used more often than guidelines without.^6^ Yet, one third of guidelines published since 2010 did not feature GItools^3^ and there is limited understanding of their effectiveness due to lack of adequate evaluation.^5^

The 2018 PCOS guideline has an exceptional record of producing accompanying GItools, including a freely available GP Tool summarising diagnosis and management, care plan template created for use in chronic disease management,^1^ and algorithms addressing screening, diagnostic/risk assessment; emotional wellbeing; lifestyle management; pharmacological treatment for non-fertility indications; and management of infertility, accompanied by significant dissemination and translation efforts. Given the vital role that GPs play in implementing the PCOS guideline recommendations and GPs’ unique understanding of primary care workplace culture, accessibility, efficiency and alignment/integration with clinical care processes and systems, it is important to seek their input on the PCOS GItools. The valuable insights provided by GPs will ensure successful implementation and ultimately will optimise the care of women and adolescents with PCOS. The aim of this study is to understand the barriers and enablers to adoption and maintenance of use of GItools developed to assist GPs in implementing recommendations from the international PCOS guideline.

## Methods

### Study design

This study used a concurrent mixed-methods design.^7^ A survey and semi-structured interviews with GPs and GPs in training were conducted across Australia (February-December 2022). Ethics approval was granted by the Western Sydney University Human Research Ethics Committee (H14733, December 2021). Development of the survey tool and interview schedule were informed by the Theoretical Domains Framework (TDF) and the Capability Opportunity Motivation – Behaviour (COM-B) theoretical model.^8^ The TDF was developed following a comprehensive review of 33 psychological and organisational theories^9,10^ and consisted of 14 domains expanding on 3 core components: Capability (having knowledge, skills, and abilities to engage in a particular behaviour), Opportunity (external factors that make a behaviour possible), Motivation (internal processes that influence decision making and behaviours).^8,10^ Both TDF and COM-B form the hub of the Behaviour Change Wheel, a method for characterising and designing behaviour change interventions. Mapping barriers and enablers onto the TDF and COM-B allows identification of intervention functions, providing a foundation on which to select intervention strategies that can effect behaviour change.

### Quantitative research—survey

#### Research instrument

The online anonymous open survey was developed using Qualtrics software (Qualtrics Ltd., Provo, UT, USA). The survey took approximately 30 min to complete and consisted of 35 items over 8 pages relating to (i) demographics; (ii) self-perceived knowledge and confidence in managing PCOS; (iii) awareness and use of the 2018 guideline and 3 GItools (GP toolkit, care plan template, and 5 algorithms); and (iv) identification of other resources used to inform management of PCOS. Most questions were provided in multiple-choice format. Self-rated knowledge and confidence in diagnosis and management was assessed using a 6-point Likert scale ranging from “excellent knowledge/confidence” to “no knowledge/confidence.” Features were enabled that prevented multiple responses from either a single internet protocol address or the same computer. Adaptive questioning was used to reduce the number and complexity of responses based on prior responses to questions on awareness of the guideline and GItools. The survey was developed by a multidisciplinary team (GPs, dietitians, women’s health experts) and was tested for usability, face validity, and piloted online. Feedback was incorporated into the final questionnaire and retested prior to use.

#### Recruitment, eligibility, and consent

The survey was open to any GP or GP in training residing within Australia. A convenience sample was recruited through advertisements in Primary Health Networks,^2^ professional networks, social media promotion, and snowball sampling from February to November 2022. Advertisements directed potential participants to a survey landing page with access to a participant information sheet. Informed consent was implied upon commencement. No incentives were offered.

#### Data analysis

Quantitative data were analysed using SPSS version 28 2021 (IBM, Armonk, NY, USA). Descriptive analyses were performed for normally distributed data (means, standard deviation), non-normally distributed data (median, interquartile ranges), and categorical variables (number, percentage). Surveys with no responses were removed from the analysis. Missing values were not replaced.

### Qualitative research—interviews

#### Research instrument

Semi-structured interviews were conducted via online video conferencing software (Zoom). Virtual interviews have been shown to provide comparable quality to face-to-face interviews^11^ and increase convenience, likelihood of participating, and facilitation of data collection from a nationally representative sample. Interviews were conducted by a dietitian (SC, PhD) and GP (CE, PhD), both female, with expertise in qualitative research methods and clinical and research experience in PCOS. There was no prior relationship between researchers and interviewees. Participants were informed of interviewers’ interests in PCOS clinical management and research. Interviews lasted 30–45 min and explored overall impression of PCOS diagnosis/management in general practice, experience with using GItools, barriers/enablers to utilising GItools, beliefs about consequences of not utilising GItools, and key motivators for using GItools. Demographic information was collected verbally. There were no other attendees present.The interview guide was developed by the research team and pilot tested by SC and CE. Field notes were taken during the interviews. Steps were taken to contribute to credibility (triangulation using multiple data sources), dependability (data saturation), confirmability (documenting steps, 10% cross-checking of transcript coding), and transferability (presenting interview quotes and participant characteristics) of data.^12^

#### Recruitment, eligibility, and consent

The survey included an invitation to participate in interviews. Purposive sampling was used, aiming for diversity in geographical location, years of experience, and genders.

#### Data collection

Interviews were audio-recorded and transcribed verbatim using a professional transcription service. Written informed consent was obtained before all interviews. Data collection continued until it was determined during team meetings that data was adequate in terms of richness and complexity in order to answer our research questions^13^ (*n* = 14). All participants were given the option to review transcripts to ensure the data accurately reflects their experience, however, all 14 participants declined. Participant feedback on findings was not sought.

#### Data analysis

We performed thematic analysis using NVivo software and a combined inductive and deductive approach. We first used open inductive coding in which codes were iteratively revised and clustered into subthemes and themes^14^ Coding and theme development were performed by SC. To ensure reliability, 10% of transcripts were independently coded by CE until consistent coding occurred. Where discrepancies took place, coded transcripts were discussed and recoded until consensus was reached.^14^ Once identified themes were discussed and agreed upon, they were deductively mapped against COM-B and TDF constructs and intervention functions.^15,16^

## Results

### Quantitative findings—survey

We received 195 responses and deleted 44 empty and 5 ineligible responses (not a GP/GP in training), leaving 146 responses in the final analysis. GPs were primarily female (90%) with mean ± SD age of 42.2 ± 9.8 years, median (IQR) of 9 (14.25) years of practice, and consulted with 1 (2) PCOS patients/month (Table 1).

**Table 1.** Characteristics of general practitioner participants in Australia (2022).

Variable	Survey (*n* = 146)^a^	Interviews (*n* = 14)
Age (years)	42.2 ± 9.8	38.5 (16.75)
Sex *n (%)*		
* *Female	90 (90)	12 (85.7)
* *Male	9 (9)	2 (14.3)
* *Prefer not to say	1 (1)	0
Years of practice	9 (14.25)	9 (10.5)
Number of PCOS patients per month	1 (2)	8 (8)
Manages Aboriginal and Torres Strait Islander patients *n* (%)	85 (91.4)	12 (85.7)
% patients CALD^b^	2 (0)	10 (20)
Geographical location *n* (%)		
* *Metropolitan	81 (55.48)	14 (100)
* *Rural	10 (6.85)	
* *Remote	3 (2.05)	
Billing *n* (%)^c^		-
* *Mixed	55 (54.5)	
* *Private	20 (19.8)	
* *Bulk	26 (25.7)	
Works in a clinic with Allied health *n* (%)	54 (53.5)	-
Works with other staff who can assist with aspects of assessment/management (e.g. nurse practitioners) *n* (%)	56 (56.6)	-
Referrals *n* (%)		-
* *Dietitian	82 (82)	
* *Exercise physiologist	61 (61)	
* *Psychologist	56 (56)	
* *Dermatologist	24 (24)	
* *Gynaecologist	63 (63)	
* *Endocrinologist	41 (41)	
* *Fertility specialist	76 (76)	
* *Other	4 (4)	

CALD, culturally and linguistically diverse; PCOS, polycystic ovary syndrome.

^a^Normally distributed data are presented as mean ± SD and non-normally distributed data are presented as medium (IQR). Data presented as *n* (%) where specified.

^b^Participants were asked to estimate the % of patients they see who are CALD and findings are summarised here as median (IQR).

^c^Bulk billing practices do not charge patients an out-of-pocket cost and instead choose to be paid reduced fees directly from the Australian government. Mixed billing practices charge out-of-pocket costs to some patients and waive out-of-pocket costs for others (e.g. for patients who are on a pension). Private billing practices charge all patients an out-of-pocket cost.

#### Awareness and use

While the majority of GPs (64%) were aware of the 2018 guideline, less than half (38%) believed they had improved their practice. The most common way of being informed about the guideline was through a professional society (44%), followed by health media (22%).

The majority of respondents were unaware of the GItools (69/125 or 55.2% for the toolkit, 82/116 or 70.7% for the algorithms, and 68/111 or 61.3% for the care plan template). Less than a third had used them in clinical practice. Of those who had used these tools, the Toolkit and Algorithms were found to be useful/very useful by 70% of GPs, while the Care Plan Template was rated as somewhat/not at all useful by 73%. The easiest resource to use was the Toolkit (82% rated as easy/very easy), while less than half of GPs rated the Algorithms (48%) and the Care Plan Template (46%) easy to use. Most GPs used the Algorithms to guide diagnosis (59%) and the Toolkit and Care Plan Template to guide management (73% and 75%, respectively) (Supplementary Table S1). Two thirds of GPs indicated they had used other resources to manage PCOS (Supplementary Table S2).

#### Barriers and enablers to utilising guideline implementation tools

The most frequent barriers were lack of awareness, lack of integration into electronic medical record systems, and difficulty locating tools. GPs also lacked time, forgot to use tools, or found them too complex to use (Table 2). Enablers included integrating resources into electronic medical record systems, making them simpler and quicker to use and more readily available online, and evidence of improved outcomes with use (Table 3).

**Table 2.** General practitioner barriers to utilising guideline implementation tools when assessing and managing polycystic ovary syndrome in Australia (2022)^a^.

Barriers	PCOS GP Toolkit *n* = 121 responses	Algorithms *n* = 116 responses	GP Care Plan Template *n* = 107 responses
Not being made aware of it	76 (62.8)	85 (73.3)	70 (65.4)
Forget to use	37 (30.6)	39 (33.6)	32 (29.9)
Cannot locate it easily	58 (47.9)	63 (54.3)	51 (47.7)
Not enough time	45 (37.2)	49 (42.2)	37 (34.6)
Too complex to use	28 (23.1)	47 (40.5)	29 (27.1)
The tool doesn’t inform my practice	12 (9.9)	10 (8.6)	15 (14)
Not integrated into my electronic medical record system (e.g. Best Practice, Medical Director)	61 (50)	48 (41.4)	66 (61.7)
No experience in how to use the tool to inform practice	18 (14.9)	19 (16.4)	7 (6.5)
No training in how to use the tool to inform practice	20 (16.5)	10 (8.6)	7 (6.5)
I don’t think it will make a difference to patient outcomes	7 (5.8)	8 (6.9)	9 (8.4)
I don’t believe there is any benefit to me from using the tool	9 (7.4)	5 (4.3)	6 (5.6)
Does not reflect what the important issues are in clinical practice	5 (4.1)	6 (5.2)	4 (3.7)
Did not find it helpful when I used it	4 (3.3)	7 (6)	4 (3.7)
Evidence from guidelines is inconsistent	4 (3.3)	5 (4.3)	4 (3.7)
Evidence from guidelines is insufficient	6 (5)	4 (3.4)	4 (3.7)
No incentive to use	7 (5.8)	7 (6)	3 (2.8)
Other GPs have not used it	1 (0.8)	3 (2.6)	3 (2.8)
No sense of practice satisfaction gained from using the tool	4 (3.3)	3 (2.6)	4 (3.7)
Other	10 (8.3)	12 (10.3)	10 (9.3)

GP, general practitioner; PCOS, polycystic ovary syndrome.

^a^Results are presented as n (%) and there was no limit to the number of answers survey respondents could select.

**Table 3.** General practitioner enablers to utilising guideline implementation tools when assessing and managing polycystic ovary syndrome in Australia (2022)^a^.

Barriers	PCOS GP Toolkit *n* = 122 responses	Algorithms *n* = 113 responses	GP care plan template *n* = 103 responses
More readily available online	67 (54.9)	68 (60.2)	43 (41.7)
Simple to use	63 (51.6)	80 (70.8)	51 (49.5)
Integrated into electronic medical record (e.g. Best Practice, Medical Director)	82 (67.2)	76 (67.3)	87 (84.5)
Reminders to use	38 (31.1)	45 (39.8)	34 (33)
Incentive to use	6 (4.9)	6 (5.3)	6 (5.8)
Training provided to use	23 (18.9)	23 (20.4)	11 (10.7)
Doesn’t take much time to use	56 (45.9)	45 (39.8)	19 (18.4)
Evidence that patients have improved outcomes after using the tool	45 (36.9)	32 (28.3)	32 (31.1)
Being more aware of the possible negative consequences of not using the tool	11 (9)	4 (3.5)	8 (7.8)
Examples provided on how to integrate the tool into practice (e.g. using knowledge gained from the PCOS GP Tool to inform patient management)	33 (27)	17 (15)	12 (11.7)
Evidence that GPs are more satisfied after using the tool	5 (4.1)	3 (2.7)	3 (2.9)
Addresses an important clinical issue	13 (10.7)	20 (17.7)	12 (11.7)
High awareness about the tool (e.g. through medical media, CPD talks)	26 (21.3)	21 (18.6)	14 (13.6)
I know that other GPs use it	13 (10.7)	8 (7.1)	4 (3.9)
Care more about the negative consequences of not using the tool	5 (4.1)	3 (2.7)	1 (1)
Develop a habit of using the tool (e.g. get into a pattern of doing it without having to think)	46 (37.7)	30 (26.5)	16 (15.5)
Feel you want to use the tool more (e.g. feel more of a sense of satisfaction)	3 (2.5)	4 (3.5)	6 (5.8)
Other	6 (4.9)	7 (6.2)	6 (5.8)

CPD, continued professional development; GP, general practitioner; PCOS, polycystic ovary syndrome.

^a^Results are presented as n (%) and there was no limit to the number of answers survey respondents could select.

### Qualitative research—interviews

Twenty-two GPs expressed interest, and 14 provided qualitative data, while the remaining 8 either did not respond to contact or were too busy to participate. Similar to survey respondents, a high proportion (86%) were female, and just over half (8/14, 57%) were aware of the GItools. Median (IQR) age was 38.5 (16.75) years with 9 (10.5) years of practice and consulting with 8 (8) PCOS women/month (Table 1).

#### Theoretical domains framework and capability opportunity motivation—behaviour model analysis

Thematic analysis revealed 9 themes and 34 subthemes (12 barriers and 22 enablers), mapped to 7 TDF domains and 5 COM-B components (Table 4). Themes are indicated in italics below.

**Table 4.** General practitioner barriers and enablers to uptake and utilisation of guideline implementation tools for polycystic ovary syndrome in Australia, mapped to the Theoretical Domains Framework and Capability Opportunity Motivation—Behaviour model (2022).

COM-B macro and micro constructs	TDF domains	Themes	Subthemes barriers	Subthemes enablers	Representative quote
Capability—psychological	Behavioural regulation	Breaking habits	Preferencing familiar resources	Keeping up-to-date with practice recommendations	I happily pull that resource up and I’ll be like yep, I know exactly what I’m looking for. Maybe it’s because I’m so familiar with it as well but that makes it easy for me to just pull it up. – P1
	Cognitive and interpersonal skills	Reflective practice	-	Identifying knowledge gaps	I think people that recognise their knowledge limitations are going to be the ones that use tools of any sort PCOS or not. Just making someone aware of their existence and availability if they aren’t able to reflect on their own learning needs, isn’t going to be helpful. – P10
Opportunity—physical	Environmental context and resources	Resource awareness	Limited awareness	Improving online visibility Integration into GP training	Yeah, probably again you don’t know what you don’t know in general practice. So if you don’t know that these tools exist, you won’t use them. – P11
		Resource accessibility	Poor navigation and layout Length does not reflect consult times Not integrated into practice software Not aesthetically aligned with women’s health	Incorporation of user-friendly features (flow diagrams, dot points, digital navigation) Compatible with practice software interface Graphics and colour scheme reflect reproductive-aged women	You need to target the GPs that are going through their training. There are often resources that different training providers will recommend. These are the resources we remember and often go to first. – P1 I think if it was hard to navigate – like it was a clunky resource. So I think a clunky resource that you can’t quickly navigate in a busy consultation isn’t going to get used. – P11 People make these beautiful PDFs and Word documents and they send it to us and it’s like, if we can’t import it into our practice software to auto populate, it’s not going to get used. – P9
		Resource relevance	Best practice isn’t always practical Recommendations are not specific to the GP context General population-level recommendations are assumed knowledge	Recommendations can be practically applied within the clinical setting Recommendations are relevant to the GP scope of practice Facilitates referrals by outlining how, when and who to refer to HAES and weight neutral approaches incorporated into lifestyle information Highlights changes in care across the lifespan	Well I’m going to refer to the dermatologist anyway, they can do the Ferriman-Gallwey score, I’m not going to. We’re not going to sit there and go through an extra five minutes of a 15-minute consult doing all of these questioners. – P9 Assessment of treatment of infertility is almost useless for GPs because we’re not allowed to use any of those medications. So that whole section may as well just focus on how to investigate and refer. – P7 I think the diagnosis and treatment of emotional wellbeing is too wordy. All GPs do this, it’s our bread and butter. They don’t need to be told how to ask about emotional wellbeing. – P5
Opportunity—social	Social influences	Practice culture	Corporate business models create negative learning environments	Registrars and female colleagues facilitate peer learning	When there’s a practice with a lot of doctors in training and especially female GPs, it tends - like we talked a lot about PCOS at our last practice. We had questions, we talked to each other, so I felt like it was well managed. Whereas at my current practice there are less registrars and we predominantly have male GPs, so PCOS is less on the radar. - P1 GPs are time poor and I think that has a lot to do with the way general practice is funded. It’s a fee for service model. Unless you’re not seeing patients, you’re not making money. You’re not on a salary to be able to go do self-education. So, it has to be in your own time and it has to be efficient. – P2 The environment that you are working in will have a big impact on uptake of anything like this. If it’s an environment that encourages ongoing learning and collegiality and professional development... Then I would say that would be a prime place where something like this would be used versus a clinic that is very business orientated. Corporate models of throughput rather than depth or breadth of care. There would be no kind of cultural push for use of anything like this because that takes more time and you are seeing less patients. – P10
		Patient-GP relationship	Disengaged patients	Proactive patients requesting more information	I guess really in a lot of instances, it’s probably more patient-driven. I think the patient is coming in asking for something or demanding something or wanting further investigation about something. That sort of prompts the GP to go into it and look into it if they don’t have the knowledge or the information to go seeking it to be able to help that patient. – P 2
Motivation—reflective	Professional role and identity Beliefs about consequences	Patient-centred care	Broad scope of practice limits depth of clinical knowledge for all relevant clinical presentations Resources are not promoted as a means to build patient trust/engagement	Facilitates GP-patient communication and co-development of management goals Designed for co-use between GP-patient Evidence-based guidelines improve patient and clinician satisfaction	This is why it’s so hard for GPs to keep up to date, because we have to deal with everything and none of them takes up very much of our time... So obviously the important thing is we listen to the patient, and work out what their specific needs are first. – P14 I guess for me, a big part of what I do in terms of managing - and this is managing any medical condition - is as much as I can try to empower the patient. So, I think having a resource that can be used to help educate the patient is quite important. – P2 For tailoring care, like setting small achievable goals, it would be good to have examples of goals that people could work towards. Like common things that the patient in front of you could try. – P3 I think just getting a clearer answer for people and an appropriate answer can be really helpful for someone that has not felt validated before. I think the number one utility for patient impact is just being heard. – P10
Motivation—automatic	Reinforcement	Incentives	-	GP Management plan	Understanding the consequences of not using these tools is important… you’d fall out of date with the current practice, you might misdiagnose, you might mistreat, and you might be not providing the best level of care that you can for your patient. – P4 I know some GPs like to do management plans because they think it helps them to earn money. It would be useful for a GP who needs some guidance as to how to fill out a chronic disease plan and meet the Medicare benefits criteria – P13

COM-B, Capability Opportunity Motivation—Behaviour; TDF, Theoretical Domains Framework.

### Capability—psychological

GPs acknowledged they often used potentially out-dated resources out of familiarity and had some difficulty with *breaking habits,* not always having systems in place to stay current with guideline iterations. GPs saw their ability to engage in *reflective practice* and accurately identify knowledge deficits as integral to improving uptake and utilisation of GItools. They recognised the importance of setting up reminder systems that alert GPs to current evidence-based resources and tools used to inform clinical practice.

*I happily pull that resource up and I’ll be like yep, I know exactly what I’m looking for. Maybe it’s because I’m so familiar with it as well but that makes it easy for me to just pull it up. – P1*

### Opportunity—physical

Lack of *resource awareness*, limited *resource accessibility*, and *resource relevance* were key barriers. Information provided was not always relevant to scope of practice (e.g. pharmacotherapy for infertility) or not practical within short consultation times (e.g. visual scale for hirsutism). Conversely, well-known information (e.g. generic population-level lifestyle management) was less valued. Similar to survey findings, the lack of integration with electronic medical record systems (in particular the Care Plan Template) was a barrier. GPs perceived the colour choice did not align with a women’s health theme.

To improve awareness, GPs suggested search engine optimisation, integration into GP training curricula and promotion on relevant social media streams and well-recognised women’s health platforms. For ease of navigation, GPs suggested flow diagrams, concise lists and dot points to summarise key information, and interactive online features for access to more detailed information when desired. The ideal length of a GItool was suggested to be a maximum of 1 double-sided A4 sheet. Content should be relevant to general practice. Clear, comprehensive, and practical guidance on pharmacotherapy regimens, dose, initiation, and maintenance of relevance were desired as were practice software reminders to use GItools.

*People make these beautiful PDFs and Word documents and they send it to us and it’s like, if we can’t import it into our practice software to auto populate, it’s not going to get used. – P9*

### Opportunity—social

Different practice business models and resulting *practice culture* were seen to influence the learning environment with some models of corporate-owned clinics perceived as more likely to discourage continuing professional development within working hours due to pressure to make a profit. Practices with a higher volume of GPs in training and newly fellowed GPs were seen to foster an environment more focussed on learning, where GPs feel less threatened to seek peer-to-peer learning. GPs in training were seen as an efficient means to disseminate knowledge within the practice on the most current resources due to their studying for Fellowship exams. Female GPs were widely recognised as being generally more interested and experienced in women’s health. The importance of the *patient-GP relationship* was highlighted whereby social pressure from patients who were willing to challenge their GP’s management strategies or request further information was a common facilitator.

### Motivation—reflective

The generalist nature of general practice made it challenging to stay abreast of changes in evidence-based recommendations for a wide range of conditions. These inevitable gaps in knowledge were seen to occasionally result in inappropriate/delayed diagnosis and suboptimal care provision.

Enablers included co-use with patients, and promotion of GItools as a means to providing *patient-centred care*, building trust, and improving compliance. GPs’ understanding of the importance of using up-to-date practice guidelines to improve patient satisfaction was seen as a core motivator for accessing evidence-based resources.

*This is why it’s so hard for GPs to keep up to date, because we have to deal with everything and none of them takes up very much of our time... So obviously the important thing is we listen to the patient, and work out what their specific needs are first. – P14*

### Motivation—automatic

Financial incentives associated with initiating a GP Management Plan were an enabler.

#### Intervention functions and components

To maximise capability for utilising GItools, GPs require *education* (provision of information) on the importance of reflective practice and *training* (attaining skills) on utilising reflective practice to identify knowledge gaps and seek appropriate resources, as well as on how to establish reminders and routines for staying current with guidelines.

Maximising opportunity requires *environmental restructuring* (changing physical and social context) and *enablement* (increasing means/reducing barriers to increase capability [beyond education and training] or opportunity [beyond environmental restructuring]), including optimising exposure, relevance, and ease of use of GItools. *Educating* patients about benefits of shared decision making, and *modelling* (providing examples of behaviours to imitate) and how to better advocate for improved care provision were also identified.

To increase motivation, *educating* and *persuading* (inducing positive feelings to stimulate action) GPs that use of GItools will improve patient satisfaction by facilitating patient-centred care can be used. *Incentives* (creating an expectation of reward) may include the additional income from creating a GP Management plan.

**Fig. 1.**
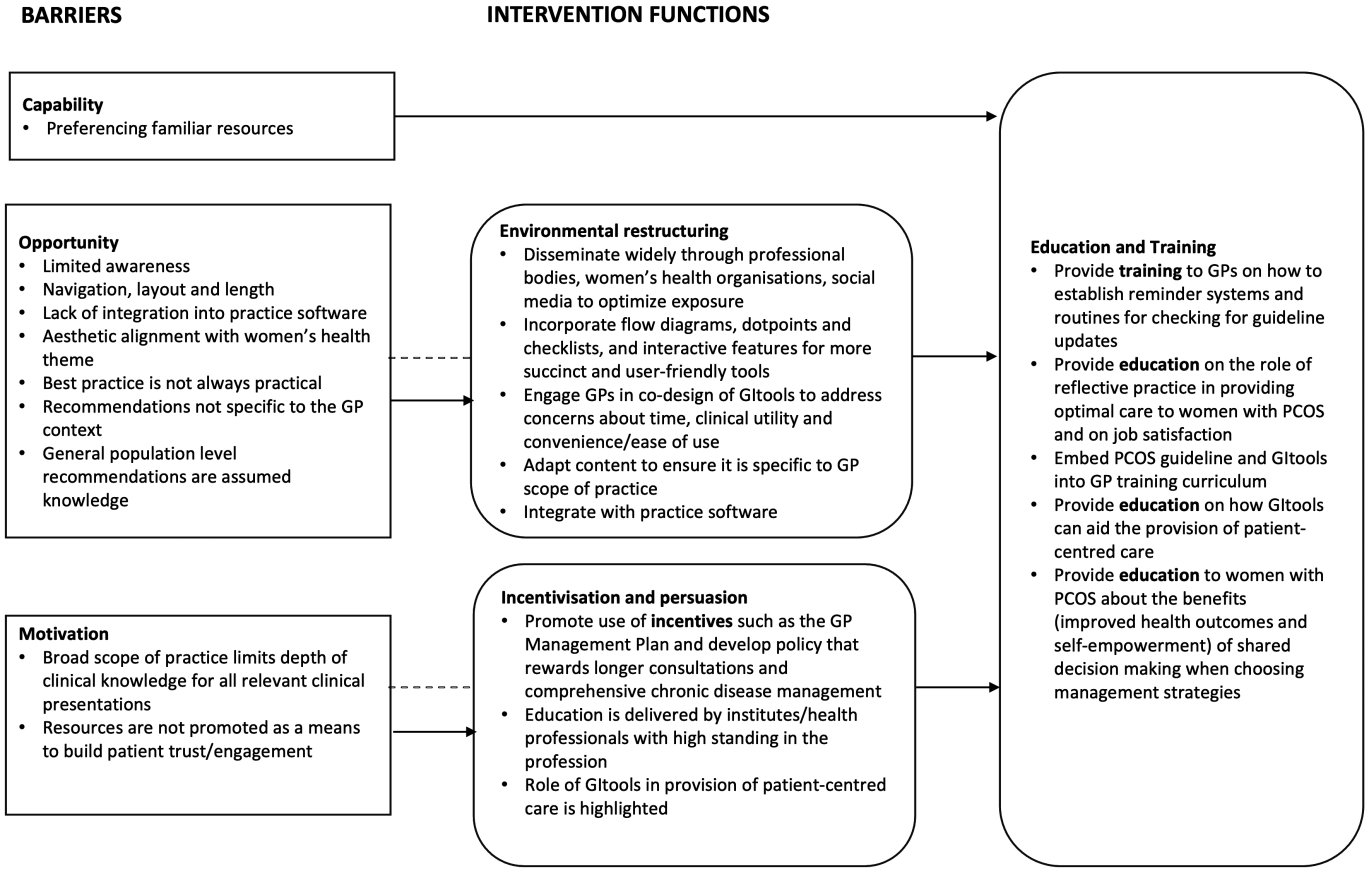
Barriers to the use of guideline implementation tools and associated intervention functions.

We identified 15 behavioural change techniques across 3 COM-B domains and intervention components to address the identified barriers and enablers (Fig. 1 and Supplementary Table S3).

## Discussion

This mixed-methods study is the first to explore how GPs engage with and utilise the PCOS GItools, using a theory-based approach to evaluate barriers, enablers, and intervention functions. Key barriers and enablers across capability (breaking habits, reflective practice), opportunity (limited awareness, impact of navigation and layout on ease of use, integration with practice software), and motivation (evidence for improved outcomes) were identified, and intervention functions were mapped. Our findings make a significant contribution to informing the implementation of guideline recommendations within the general practice setting both within PCOS management and more broadly.

Our findings align with previous research^17–19^ identifying lack of awareness of guidelines and GItools as the largest barrier identified by GPs to guideline uptake. Despite extensive and targeted translation efforts by the guideline team, including presentations, publications, and podcasts with wide reach within the Australian GP community, the level of awareness remained below the desired level. A unique enabler for clinical guideline implementation identified in our study is the role of reflective practice. This has not been reported previously,^17,20,21^ which is surprising, given it is a learnt skill that aids GPs to identify knowledge gaps and initiate professional development opportunities to improve practice.^22,23^ Reflective practice can be fostered with regular monitoring/feedback systems, such as practice audits and supervision schedules with feedback, previously highlighted as important enablers for guideline uptake and adherence.^24–27^

Implementation of clinical practice guidelines is affected by multiple factors, including limited organisational infrastructure impacting time provision and resource allocation,^28–31^ lack of policy support for funding systems encouraging long-term coordinated care,^28–31^ scepticism about guideline benefits and relevance,^28,31^ and patient non-compliance and unrealistic expectations.^32^ There are major systems-level barriers contributing to the failure to use GItools in Australia, including time pressures due to reduced rebates for general practice consultations resulting in the emergence of “six minute medicine.” GPs in Australia are mostly self-employed, which limits capacity to engage in non-clinical time to seek out new guidelines and use GItools. GItools which support implementation (e.g. decision-making aids), and evaluation (e.g. audit instructions), may assist in overcoming some of these barriers.^6,33,34^

Interventions that improve opportunity via restructuring the physical and social environment are likely to produce the most impactful and rapid benefits to guideline implementation. Key strategies include improving awareness and access to GItools, integration into electronic medical record systems and involving GPs in co-design of lean tools such as flow charts and GItools that promote co-use with patients to facilitate real-world translation. Health professionals across different levels of care have expressed a need to optimise translation of best-practice recommendations into real-world care.^18,24,35–37^ Engaging end-users in co-design can optimise the compatibility of GItools with the realities of clinical practice.^38,39^ Co-design ensures recommendations are not only based on evidence for therapeutic effects but also consider practical implementation factors such as cost, time, clinical utility, and convenience.^40^

Many of these strategies can be immediately implemented by guideline development groups, however, solutions that involve policy and organisational changes at a national level are required to ensure improvements to guideline uptake and utilisation are sustained longer-term. Significant cost barriers such as those relevant to integration into existing electronic health systems may limit feasibility. Implementation also requires a coordinated and collaborative effort with key stakeholders, and varying levels of stakeholder engagement may limit success.

A strength of our study is the use of triangulation from qualitative and quantitative data to enhance the validity and credibility of findings and allow the exploration of diverse perspectives to create an in-depth understanding of the research question. Other strengths include a rigorous theory-based approach to inform data collection and analysis. However, our findings are representative of a small proportion (0.45%) of Australian GPs, and relevance to other countries with different health care structures is less clear. These findings also likely reflect the views of GPs with a greater interest in PCOS, and hence, the sample may disproportionately reflect the experience of female GPs living in metropolitan areas.

## Conclusion

These findings highlight inherent barriers within the Australian healthcare system that hinder GPs from integrating evidence and will underpin behaviour change interventions to assist GPs in effectively utilising guidelines in clinical practice, therefore helping to minimise variations in care. While individual interventions leverage small-scale changes, changes at organisational and policy levels are needed to address the identified barriers. Our findings will have a direct influence on forthcoming guideline translation initiatives, enhancing their feasibility, adoption and rapid integration into practice, both in PCOS and with application to other clinical guidelines more broadly.

## Supplementary material

Supplementary material is available at *Family Practice* online.

cmae007_suppl_Supplementary_Tables

cmae007_suppl_Supplementary_Checklist

cmae007_suppl_Supplementary_Strobe_Checklist

## Data Availability

Data are available from the research team upon reasonable request.

## Footnotes

1 Patients with chronic medical conditions in Australia may be eligible for services under the General Practitioner Management Plan and Team Care Arrangements items. These involve the development of a care plan to coordinate care and attract a consultation rebate. Care plans outline healthcare needs, health problems, management goals and actions, and treatments and services needed.

2 Primary Health Networks in Australia are independent organisations working to streamline health services and better coordinate care.
