# Supplementary material for: Translating evidence into practice in primary care management of adolescents and women with polycystic ovary syndrome: a mixed-methods study
Source: Fam Pract. 2024 Mar 4;41(2):175–84. doi: 10.1093/fampra/cmae007 (PMC11017779; doi:10.1093/fampra/cmae007)
Supplement: cmae007_suppl_Supplementary_Tables [file cmae007_suppl_supplementary_tables.pdf]

Supplementary data

Table S1 Awareness and use of the 2018 PCOS Evidence Based Guidelines and its associated tools (survey)

Variable	Response n (%)
**Are you aware of the 2018 International Evidence based Guideline for the assessment and management of PCOS? (n=145 responses)**	
Yes	93 (64.1)
**How were you informed of the 2018 International Evidence based Guideline for the assessment and management of PCOS? (n=90 responses, could select all that apply)**	
Professional society (newsletters, education events, communication channels)	40 (44.4)
General media (television, radio, web-based news)	6 (6.7)
Health media (television, radio, web-based news)	20 (22.2)
Conference	13 14.4)
Other – *including registrar and fellowship training/exam preparation, self-acquired through internet searching/Google, social media (e.g. professional Facebook groups), colleagues, involvement in research studies, diagnostic reports (e.g. radiology reports for Polycystic ovarian morphology on ultrasound)*	19 (21.1)
**Do you think the 2018 PCOS Guideline and its associated resources (PCOS GP Toolkit, Algorithms and GP Care Plan Template) have improved your practice? (n=105 responses)**	
Yes	40 (38.1)
No	9 (8.6)
Not sure	10 (9.5)
Not applicable (I don't use them)	46 (43.8)
**How often do you use the PCOS GP Toolkit in routine practice? (n=125 responses)**	
Frequently (almost or all patients with PCOS where relevant)	9 (7.2)
Sometimes (around 1 out of 5 patients)	16 (12.8)
Rarely (around 1 out of 10 patients or less)	11 (8.8)
Aware but have never used	18 (14.4)
Unaware of resource	69 (55.2)
Other	2 (1.6)
**How did you use the PCOS GP Toolkit? (n=33 responses, could select all that apply)**	
Make a diagnosis	15 (45.5)
Guide management	24 (72.7)
Guide risk assessment	7 (21.2)
Read about it in own time/CPD	7 (21.2)
Other	1 (3.0)
**How useful have you found the PCOS GP Toolkit? (n=34 responses)**	
Extremely useful	6 (17.6)
Very useful	9 (26.5)
Useful	15 (44.1)
Somewhat useful	4 (11.8)

Not useful	0
**How easy was the PCOS GP Toolkit to use in clinical practice? (n=33 responses)**	
Very easy	8 (24.2)
Easy	19 (57.6)
Neutral	6 (18.2)
Difficult	0
Very difficult	0
**How often do you use the algorithms in routine practice? (n=116 responses)**	
Frequently (almost or all patients with PCOS where relevant)	5 (4.3)
Sometimes (around 1 out of 5 patients)	7 (6)
Rarely (around 1 out of 10 patients or less)	11 (9.5)
Aware but have never used	9 (7.8)
Unaware of resource	82 (70.7)
Other	2 (1.7)
**How did you use the algorithms? (n=22 responses, could select all that apply)**	
Make a diagnosis	13 (59.1)
Guide management	9 (40.9)
Guide risk assessment	4 (18.2)
Read about it in own time/CPD	7 (31.8)
Other	1 (4.5)
**How useful have you found the algorithms? (n=21 responses)**	
Extremely useful	1 (4.8)
Very useful	7 (33.3)
Useful	8 (38.1)
Somewhat useful	4 (19)
Not useful	1 (4.8)
**How easy were the algorithms to use in clinical practice? (n=21 responses)**	
Very easy	3 (14.3)
Easy	7 (33.3)
Neutral	9 (42.9)
Difficult	2 (9.5)
Very difficult	0
**How often do you use the GP Care Plan Template in routine practice? (n=111 responses)**	
Frequently (almost or all patients with PCOS where relevant)	9 (8.1)
Sometimes (around 1 out of 5 patients)	13 (11.7)
Rarely (around 1 out of 10 patients or less)	9 (8.1)
Aware but have never used	9 (8.1)
Unaware of resource	68 (61.3)
Other	3 (2.7)
**How did you use the GP Care Plan Template? (n=25 responses, could select all that apply)**	
Make a diagnosis	2 (8)
Guide management	19 (76)
Guide risk assessment	2 (8)
Read about it in own time/CPD	2 (8)
Other	4 (16)
**How useful have you found the GP Care Plan Template? (n=26 responses)**	
Extremely useful	1 (3.8)
Very useful	6 (23.1)

Useful	0
Somewhat useful	9 (34.6)
Not useful	10 (38.5)
**How easy was the GP Care Plan Template to use in clinical practice? (n=26 responses)**	
Very easy	3 (11.5)
Easy	9 (34.6)
Neutral	12 (46.2)
Difficult	2 (7.7)
Very difficult	0

Abbreviations: GP, general practitioner.

Table S2 Other resources used to guide assessment and management of PCOS (survey)

**Are there any other resources you use? (n=78 responses)**	
Yes	53 (67.9)
**How did you find out about these resources? (n=50 responses, could select all that apply)**	
RACGP CPD portal	7 (14)
GP learning portal	3 (6)
Organisation email/social media	13 (26)
Primary Health Network communication	7 (14)
Medical Media advertisement (e.g. Ausdoc, Medical Observer)	3 (6)
Conference	9 (18)
General media	1 (2)
Word of mouth	20 (40)
Other – *including registrar and fellowship training/exam preparation, self-acquired through internet searching/Google, in-service training and PCOS management protocols developed by their practice clinic*	17 (34)

Abbreviations: CPD, conditioned professional development; GP, general practitioner; RACGP, Royal Australian College of General Practitioners.

Table S3 Mapping identified barriers and enablers to intervention functions according to the COM-B model

**COM-B construct**	**TDF**	**Barriers**	**Enablers**	**Intervention functions**	**BCT**	**Intervention components**[a]
Psychological Capability	Behavioural regulation	• Preferencing familiar resources	• Keeping up-to-date with practice recommendations	• Training	• Instruction on how to perform a behaviour	• Provide training to GPs on how to establish provider reminder systems and routines for checking guideline updates and to ensure they are utilising the most relevant tools to guide practice
	Cognitive and interpersonal skills	-	• Identifying knowledge gaps	• Education<br>• Training	• Information about health consequences<br>• Information about emotional consequences<br>• Instruction on how to perform a behaviour<br>• Behavioural practice/rehearsal	• Provide education to GPs on the role of reflective practice in providing optimal care to patients with PCOS<br>• Provide education to GPs on the relationship between reflective practice and job satisfaction<br>• Embed PCOS guideline and GItools into GP training curriculum
Physical opportunity	Environmental context and resources	• Limited awareness<br>• Can be challenging to navigate	• Improving online visibility<br>• Integration into GP training	• Environmental restructuring	• Restructuring the physical environment<br>• Restructuring the social environment	• Use widespread dissemination via online platforms that are frequently used by GPs, including well recognised women's health organizations, professional

		• Length does not reflect consult times<br>• Not integrated into practice software<br>• Not aesthetically aligned with women's health<br>• Best practice isn't always practical<br>• Recommendations are not specific to the GP context<br>• General population level recommendations are assumed knowledge	• Incorporation of user-friendly features (flow diagrams, dot points, digital navigation)<br>• Compatible with practice software interface<br>• Graphics and colour scheme reflect reproductive aged women<br>• Recommendations can be practically applied within the clinical setting<br>• Recommendations are relevant to the GP scope of practice<br>• Facilitates referrals by outlining how, when		• Adding objects to the environment<br>• Credible source	bodies, professional development/training services, social media and other prominent GP communication channels<br>• Incorporate flow diagrams, checklists/dot-points and digital/interactive features (e.g. clickable links to further information/resources for novice GPs) to reduce the length and improve the usability of implementation tools<br>• Incorporate colour schemes and graphics that are consistent with a women's health theme<br>• Involve GPs in co-design of implementation tools to ensure they consider practical aspects such as time, clinical utility and convenience<br>• Adapt content in implementation tools to ensure it is specific to GPs role/responsibilities within the broader multidisciplinary care team, and includes details on when/who/how to refer to other health professionals

			and who to refer to<br>• HAES and weight neutral approaches incorporated into lifestyle information<br>• Highlights changes in care across the lifespan			• Link guidelines and implementation tools to clinical management software (i.e. prompting GPs to utilise guidelines and tools when making a PCOS diagnosis, developing management strategies and educating patients)
Social opportunity	Social influences	• Corporate business models create negative learning environments	• Registrars and female colleagues facilitate peer learning<br>• Proactive patients requesting more information	• Environmental restructuring<br>• Education<br>• Modelling<br>• Enablement<br>• Incentivisation	• Restructuring the physical environment<br>• Restructuring the social environment<br>• Social support (practical)<br>• Information about health consequences<br>• Information about emotional consequences<br>• Material and social reward<br>• Reward alternative behaviour	• Appoint working groups and/or leadership roles within practice clinics that can assign financial and/or physical resources to support professional development activities<br>• Develop GItools for implementation support, outlining the necessary resources (human, infrastructure or funding), instructions or processes<br>• Promote the use of incentives such as the Chronic Disease Management Plan to encourage GItool use<br>• Develop policy that rewards longer consultations and

					• Demonstration of a behaviour<br>• Instruction on how to perform the behaviour<br>• Identification of self as role model	comprehensive chronic disease management rather than short episodes of acute care<br>• Provide education to women with PCOS about the benefits (improved health outcomes and self-empowerment) of shared decision making when choosing management strategies<br>• Encourage patients to advocate for optimal care and act as role models for others
Reflective motivation	Professional role and identity	• Broad scope of practice limits depth of clinical knowledge for all relevant clinical presentations<br>• Resources are not promoted as a means to build patient trust/engagement	• Facilitates GP-patient communication<br>• Examples of how to tailor and co-develop management goals<br>• Designed for co-use between GP-patient	• Persuasion<br>• Education<br>• Environmental restructuring	• Information about health consequences<br>• Information about social and environmental consequences<br>• Credible source<br>• Restructuring the physical environment	• Provide education on how guideline implementation tools can aid tailoring of management strategies, encorage effective provider-patient communication and facilitate patient education to promote patient centred care<br>• Education (as per above) should be incorporated into existing education programmes delivered by institutes/health professionals with a high-standing within the profession

						• Develop implementation tools that are designed for co-use by the GP (as a simple/quick checklist for diagnosis and management) and patients (as a lay summary for patient education)
	Beliefs about consequences	-	• Evidence-based guidelines improve patient satisfaction	• Education<br>• Persuasion	• Information about health consequences<br>• Information about emotional consequences<br>• Credible source	• Provide education to GPs on the role of evidence-based guidelines and tools in providing optimal care to patients with PCOS<br>• Education (as per above) should be promoted by trusted professional bodies and women's health organisations through which guideline promotion/dissemination takes place
Automatic motivation	Reinforcement	-	• Enhanced Primary care plan	• Incentivisation	• Material incentive (behaviour)	• Leverage financial incentive to initiate Enhanced Primary care plans to women with PCOS to motivate GPs to access tools that facilitate efficient diagnose and development of management strategies<br>• Develop GItools for evaluation support, outlining audit tools and measures for guideline compliant practice

[a] *Considers policy categories needed to support the delivery of the intervention function (i.e. communication/marketing, guidelines, fiscal measures, regulation, legislation, environmental/social planning and service provision)*

Abbreviations: COM-B, Capability Opportunity Motivation – Behaviour; TDF, Theoretical Domains Framework.
